# Thiazolidinediones and the risk of asthma exacerbation among patients with diabetes: a cohort study

**DOI:** 10.1186/1710-1492-10-34

**Published:** 2014-07-03

**Authors:** Seppo T Rinne, Laura C Feemster, Bridget F Collins, David H Au, Mark Perkins, Christopher L Bryson, Thomas G O’Riordan, Chuan-Fen Liu

**Affiliations:** 1 Health Services Research and Development, VA Puget Sound Health Care System, Department of Veterans Affairs, 1100 Olive Way Suite 1400, 98104-3801 Seattle, WA, USA; 2 Department of Pulmonary and Cri Care, University of Washington, Seattle, WA, USA; 3 Division of General Internal Medicine, Department of Medicine, University of Washington, Seattle, WA, USA; 4 Gilead Sciences, Inc., Seattle, WA, USA; 5 Department of Health Services, University of Washington, Seattle, WA, USA

**Keywords:** Thiazolidinediones, Peroxisome proliferator-activated receptors, Glitazones, Asthma, Cohort study

## Abstract

**Background:**

Thiazolidinediones are oral diabetes medications that selectively activate peroxisome proliferator-activated receptor gamma and have potent anti-inflammatory properties. While a few studies have found improvements in pulmonary function with exposure to thiazolidinediones, there are no studies of their impact on asthma exacerbations. Our objective was to assess whether exposure to thiazolidinediones was associated with a decreased risk of asthma exacerbation.

**Methods:**

We performed a cohort study of diabetic Veterans who had a diagnosis of asthma and were taking oral diabetes medications during the period of 10/1/2005 – 9/30/2006. The risk of asthma exacerbations and oral steroid use during 10/1/2006 – 9/30/2007 was compared between patients who were prescribed thiazolidinediones and patients who were on alternative oral diabetes medications. Multivariable logistic regression and negative binomial regression analyses were used to characterize this risk. A sensitivity analysis was performed, restricting our evaluation to patients who were adherent to diabetes therapy.

**Results:**

We identified 2,178 patients who were on thiazolidinediones and 10,700 who were not. Exposure to thiazolidinediones was associated with significant reductions in the risk of asthma exacerbation (OR = 0.79, 95% CI, 0.62 – 0.99) and oral steroid prescription (OR = 0.73, 95% CI 0.63 – 0.84). Among patients who were adherent to diabetes medications, there were more substantial reductions in the risks for asthma exacerbation (OR = 0.64, 95% CI 0.47 – 0.85) and oral steroid prescription (OR = 0.68, 95% CI 0.57 – 0.81).

**Conclusions:**

Thiazolidinediones may provide a novel anti-inflammatory approach to asthma management by preventing exacerbations and decreasing the use of oral steroids.

## Introduction

Asthma is a major cause of morbidity in the Unites States, affecting 22 million people and annual costs of $56 billion [1]. Much of this cost is related to the burden of acute exacerbations, which leads to approximately 1.8 million emergency room visits and 497,000 hospitalizations each year [2]. Prevention of asthma exacerbations typically relies on the use of inhaled corticosteroids to reduce chronic airway inflammation [3]. However, many patients find inhalers difficult to use and non-adherence to inhaled corticosteroids is common [4]. Furthermore, 5-10% of patients with severe asthma receive sub-optimal control even with proper use of inhaled corticosteroids [5]. Therefore, a major focus of drug development is discovering new ways to decrease airway inflammation and prevent asthma exacerbations.

Thiazolidinediones (TZDs), such as pioglitazone and rosiglitazone, are oral diabetes medications that have also been shown to have potent anti-inflammatory properties [6]. These medications act as selective agonists to the peroxisome proliferator-activated receptor gamma (PPARγ) [7]. PPARγ regulates gene expression by forming a heterodimer with the retinoid X receptor and binding DNA at the promoter region of targeted genes [8]. PPARγ activation inhibits inflammatory cytokine production and release [9]. Exposure to TZDs in animal models of asthma is associated with decreased airway inflammation and improved physical condition [10-18]. In humans, few studies have examined the effect of TZDs on asthma, and those that have been performed have focused on improvements in pulmonary function and airway hyperresponsiveness [19-21]. There are no clinical studies on the impact of TZDs on asthma exacerbations or oral steroid use.

Our primary objective was to examine how exposure to TZDs is associated with asthma exacerbation among diabetic patients. We also assessed the relationship between TZD exposure and oral steroid prescriptions among this patient population.

## Methods

### Design

We performed a cohort study of US Veterans receiving healthcare at Veterans Affairs (VA) medical facilities. Information was collected from electronic medical records of all VA service users. Baseline characteristics and TZD use were assessed during the 2006 fiscal year (10/1/2005 – 9/30/2006), and outcomes were assessed during the 2007 fiscal year (10/1/2006 – 9/30/2007). The study was approved by the VA Puget Sound Institutional Review Board (#01456).

### Study cohort

Study participants were identified by having two or more ICD-9 diagnoses of diabetes and having received at least two prescriptions for an oral antihyperglycemic medication (TZDs, sulfonylurea, or metformin) during the baseline year. Patients with co-existing asthma were identified based on outpatient and inpatient criteria. Outpatient criteria included having two or more outpatient visits with an ICD-9 code for asthma or having one outpatient visit with a diagnosis of asthma and a prescription for albuterol. Inpatient criteria included having a discharge diagnosis of asthma.

Exposure to TZD was defined as having filled a prescription for TZDs on two or more occasions during the baseline year. The majority of TZD patients in our study were prescribed rosiglitazone (96%), which was on the medication formulary at the VA during the study period; only 4% of TZD patients were prescribed pioglitazone. Patients who were exposed to TZDs could have also been prescribed other oral diabetes medications. The reference comparison was having filled a prescription for a non-TZD oral antihyperglycemic medication (either sulfonylurea or metformin) on two or more occasions during the baseline year. We chose to use a heterogeneous non-TZD comparison group in order to emphasize the impact of TZDs in asthma. We did perform additional analyses comparing TZDs to metformin use and sulfonylurea use separately and found similar results. Patients were excluded from the study if they filled only one prescription for TZDs during the baseline year.

Covariates included baseline patient characteristics, comorbidities, and current respiratory medication use. The severity of diabetes was measured by the Diabetes Severity Index score [22]. Overall patient health care burden was further evaluated using Diagnostic Cost Group scores [23]. Information on the number of asthma exacerbations and prescriptions for respiratory medications in the baseline was also recorded.

### Study outcomes

The primary outcome was asthma exacerbation, including inpatient or outpatient exacerbations during the follow-up year. Inpatient asthma exacerbations were defined by a primary ICD-9 discharge diagnosis of asthma or having a primary ICD-9 discharge diagnosis of acute respiratory failure and a secondary diagnosis of asthma. Outpatient asthma exacerbations included a primary clinic diagnosis of asthma accompanied by a prescription for oral steroids within 2 days of the visit. Due to the low prevalence of asthma exacerbations, inpatient and outpatient exacerbations were grouped together for the analysis. A secondary outcome was any oral steroid prescription. All patients were monitored for outcomes during the entire follow up year (10/1/2006 – 9/30/2007). We separated measurements of the exposure (during the baseline year) and the outcome (during the follow-up year) in order to limit the potential for immortal time bias [24].

### Statistical analysis

All statistical analyses were performed using Stata 11.2 [25]. Logistic regression was used to analyze the odds of any asthma exacerbation and any prescription of an oral steroid prescription during the follow-up year. Results were reported using odds ratios (OR) and 95% confidence intervals (CI). Negative binomial regression was used to evaluate the association of TZDs with the expected number of events. Results were reported as incidence rate ratios (IRR), which were derived by dividing the expected number of events among the TZD group by the expected number of events among the non-TZD group. For example, an IRR of 0.6 indicates that the TZD group has a 40% lower number of events than expected in the non-TZD group.

A sensitivity analysis was performed on patients who were most adherent to diabetes medications. Adherence was based on medication possession ratios which were calculated as Refill Compliance (ReComp) scores for the six months prior to the outcome period [26]. Patients were only included in the sensitivity analysis if they had a ReComp score of at least 0.8.

## Results

**Figure 1:**
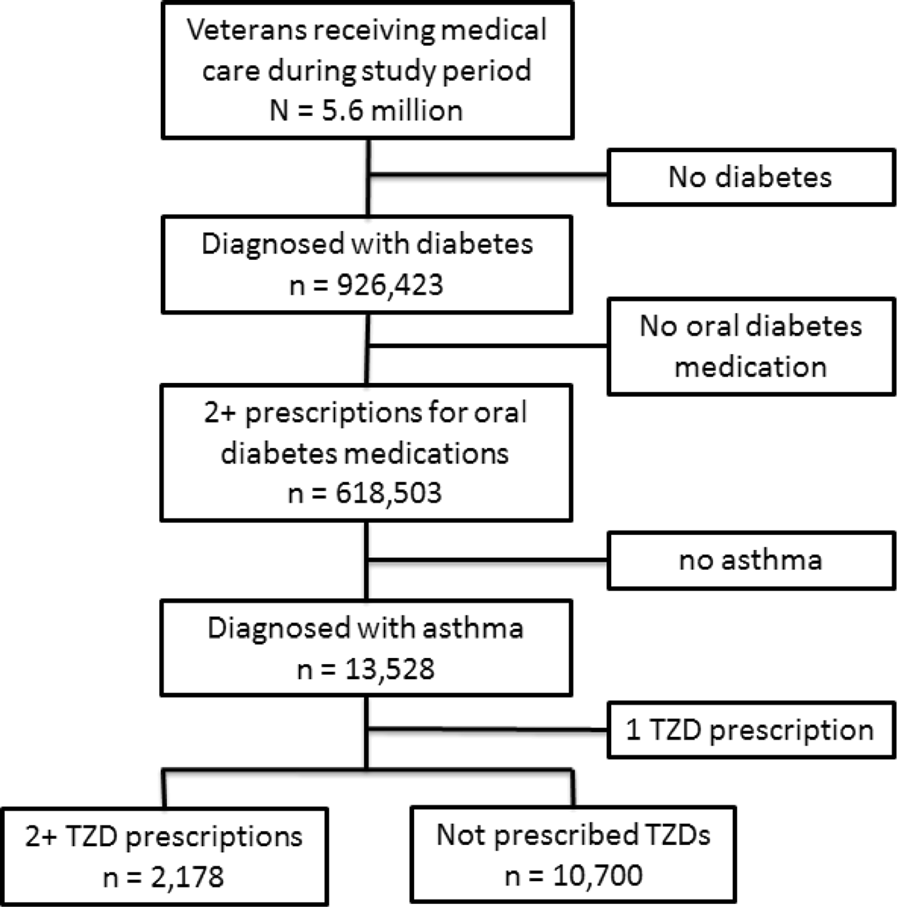
Study selection.

We identified 13,528 diabetic Veterans who had two or more prescriptions for oral diabetic medications and had been diagnosed with asthma (Figure 1). Of these, 2,178 patients had received two or more prescriptions for TZDs during the baseline year, while 10,700 did not receive any prescription for TZDs. Patient attrition was low in our study with 94.7% of patients had at least one clinic visit during the follow-up year (mean = 5.76 visits, SD = 5.73). Censorship was not different between the two study groups (5.7% of TZD patients 5.2% of non-TZD patients were seen in the VA during the follow up year).

Patients in the TZD group were more likely to be younger, nonwhite, and have more primary care visits when compared with the non-TZD group (Table 1). These patients also had more severe diabetes and had a higher diagnostic cost group score. While patients in the TZD group had a lower prevalence of atrial fibrillation, heart failure, and substance abuse, they had a higher prevalence of coronary artery disease. The TZD group had fewer asthma exacerbations in the year prior to the study and were prescribed fewer short-acting beta agonists, fewer inhaled corticosteroids, and fewer long-acting beta agonists. In contrast, they had more prescriptions for leukotriene modifiers.

**Table 1:** Baseline characteristics of patients in the TZD and non-TZD groups

**Patient characteristics**	**TZD n = 2,178**	**Non-TZD n = 10,700**	**p-value**
Age (mean/SD)	64.0 (10.4)	64.8 (11.1)	<0.01
Female (%)	6.9	7.6	0.28
Race			
White (%)	59.9	64.5	<0.01
Nonwhite (%)	21.0	18.7	0.01
Race unknown (%)	19.1	16.8	0.01
VA Freecare (%)	59.0	57.1	0.12
Distance to VA (mean/SD)	24.8 (31.4)	24.7 (30.0)	0.93
Physician encounters (mean/SD)	7.0 (5.7)	6.6 (5.6)	0.01
Prior exacerbations (mean/SD)	0.8 (1.6)	0.9 (1.7)	0.04
Diabetes severity index (mean/SD)	1.5 (1.6)	1.2 (1.4)	<0.01
Diagnostic cost group (mean/SD)	1.12 (0.7)	1.07 (0.7)	0.01
**Comorbidities**			
Atrial fibrillation (%)	6.0	8.0	0.02
Congestive heart failure (%)	6.0	7.5	0.01
Coronary artery disease (%)	22.5	20.5	0.05
Drug or alcohol abuse (%)	7.5	11.0	<0.01
Depression (%)	15.3	14.6	0.37
**Medications**			
Short acting β-agonist (mean/SD)	5.6 (6.5)	6.0 (6.6)	0.01
Inhaled corticosteroid (%)	47.0	51.7	<0.01
Leukotriene modifier (%)	28.5	25.9	0.01
Long acting β-agonist (%)	38.0	40.5	0.03

### Asthma exacerbations

In the unadjusted analysis (Table 2), patients in the TZD group were less likely to experience at least one exacerbation (inpatient or outpatient) compared to those in the non-TZD group (4.7% versus 5.9%, p = 0.03). There was no significant difference in the total number of exacerbations between the two groups (0.08 for the TZD group versus 0.10 for the non-TZD group, p = 0.06).

**Table 2:** Asthma exacerbations and oral steroid prescriptions among the TZD and non-TZD groups

**Event**	**TZD**	**Non-TZD**	**p-value**
Patients with an exacerbation* (%)	4.7	5.9	0.03
# of exacerbations (mean/SD)	0.08 (0.41)	0.10 (0.54)	0.06
Patients with a steroid prescription (%)	15 · 1	19.4	<0.01
# of steroid prescriptions (mean/SD)	0.35 (1.32)	0.50 (1.66)	<0.01

*Includes both inpatient and outpatient exacerbations.

After controlling for potential confounding factors (Table 3), there was a significantly decreased risk for any asthma exacerbations among patients in the TZD group (adjusted OR of 0.79 (95% CI 0.62 – 0.99), indicating patients in the TZD group had a 21% decreased odds of experiencing at least one asthma exacerbation in the study period. There was no significant difference in the expected incidence of exacerbations in the TZD group (adjusted IRR of 0.81, 95% CI 0.64 – 1.02).

**Table 3:** Association of TZDs with asthma exacerbations and steroid prescriptions in multivariable models^*^

**Event**	**Any event**^**†**^		**Number of events**^**‡**^	
	**OR**	**95%CI**	**IRR**	**95%CI**
Asthma exacerbation	0.79	0.62 – 0.99	0.81	0.64 – 1.02
Steroid prescription	0.73	0.63 – 0.84	0.68	0.58 – 0.80

*Adjusted for age, gender, race, free care, distance to VA facility, number of primary care encounters, Diagnostic Cost Group score, Diabetes Severity Index score, prior year exacerbations, atrial fibrillation, heart failure, ischemic heart disease, alcohol or drug abuse, and depression, respiratory medications.

^†^Logistic regression with outcome being presence of asthma exacerbation or steroid prescription. Results are presented in odds ratios (OR) and 95% confidence intervals (CI).

^‡^Negative binomial regression with outcome being number of asthma exacerbations or number of steroid prescriptions. Results are presented in incidence rate ratios (IRR) and 95% confidence intervals.

### Oral steroid prescriptions

In the unadjusted analysis, the TZD group was less likely to receive a prescription for oral steroids than the non-TZD group (15.1% versus 19.4%, p < 0.01). On average, the TZD group also received fewer prescriptions for steroids compared to the non-TZD group (0.35 versus 0.50, p < 0.01).

After controlling for potential confounding, there was a significantly decreased risk for at least one oral steroid prescription among the TZD group (adjusted OR = 0.73, 95% CI 0.63 – 0.84). Patients exposed to TZDs also had a significantly lower expected number of steroid prescriptions (adjusted IRR = 0.68, 95% CI 0.58 – 0.80). This indicates that the expected number of oral steroid prescriptions in the TZD group is 32% lower than the expected number for the non-TZD group.

### Sensitivity analysis

Restricting our analysis only to patients who were adherent to oral diabetes medications (with ReComp scores of at least 0.8) yielded more pronounced results (Table 4). There were 1,474 patients who were adherent to therapy in the TZD group and 8,208 patients who were adherent to therapy in the non-TZD group. In this analysis, patients in the TZD group had a significantly lower risk of an asthma exacerbation (adjusted OR = 0.64, 95% CI 0.47 – 0.85). The expected incidence of asthma exacerbations was also significantly lower in the TZD group (adjusted IRR = 0.65, 95% CI 0.48 – 0.88). Patients in the TZD group had a lower risk of having been prescribed any oral steroid (adjusted OR = 0.68, 95% CI 0.57 – 0.81). Similarly, these patients had a lower expected number of steroid prescriptions (adjusted IRR = 0.65, 95% CI 0.54 – 0.79).

**Table 4:** Sensitivity analysis with multivariable models^*^to evaluate the association of TZDs with asthma exacerbations and steroid prescriptions among patients who were most adherent to oral diabetes therapy (ReComp scores ≥ 0.8)

**Event**	**Risk of any event**^**†**^		**Risk of multiple events**^**‡**^	
	**OR**	**95%CI**	**IRR**	**95%CI**
Asthma Exacerbation	0.64	0.47 – 0.85	0.65	0.48 – 0.88
Steroid prescription	0.68	0.57 – 0.81	0.65	0.54 – 0.79

*Adjusted for age, gender, race, free care, distance to VA facility, number of primary care encounters, Diagnostic Cost Group score, Diabetes Severity Index score, prior year exacerbations, atrial fibrillation, heart failure, ischemic heart disease, alcohol or drug abuse, and depression, respiratory medications.

^†^Logistic regression with outcome being presence of asthma exacerbation or steroid prescription. Results are presented in odds ratios (OR) and 95% confidence intervals (CI).

^‡^Negative binomial regression with outcome being number of asthma exacerbations or number of steroid prescriptions. Results are presented in incidence rate ratios (IRR) and 95% confidence intervals.

## Discussion

This is the first clinical study examining the association of TZDs with asthma exacerbations and steroid prescriptions. Consistent with prior studies showing that TZDs have potent anti-inflammatory effects, we found significant reductions in asthma exacerbations and oral steroid prescriptions among patients who were exposed to TZDs. This study provides new evidence that TZDs may have a role in the future treatment of asthma among patients with diabetes.

Prevention of asthma exacerbations has been a cornerstone of guidelines on asthma management [3]. First line therapies target decreasing airway inflammation through the use of inhaled corticosteroids, which reduce the risk of asthma exacerbation by more than 50% [27]. However, inhaled corticosteroids are not always effective, and despite having less systemic distribution than oral steroids, they are still associated with multiple adverse effects [28]. While many patients in our study were already on inhaled corticosteroids, exposure to TZDs was associated with a further reduction in the risk for exacerbation.

Our findings demonstrating the beneficial effect of TZDs on asthma have a plausible biological mechanism. TZDs are agonists to PPARγ and are involved in a variety of biological functions, including the inflammatory response [29]. Activation of PPARγ inhibits the production and release of cytokines and cell survival factors involved in inflammation [30]. A study on human airway smooth muscle cells found PPARγ agonists were more effective at inhibiting inflammatory cytokine release than corticosteroids [31]. Animal models of asthma that are exposed to TZDs have consistently demonstrated reduced airway inflammation, mucus production, and airway hyperresponsiveness [12-15]. A study of the effect of ciglitazone on a murine model of asthma further demonstrated inhibition of airway smooth muscle remodeling [14]. This body of literature identifies a potential role for TZDs in asthma management.

Ours is the first large study looking at clinical outcomes among patients with asthma, and our findings are consistent with prior studies that showed improvements in pulmonary function and bronchoconstriction among asthmatic patients. Case reports of two diabetic patients with asthma found improvement in respiratory symptoms after initiation of pioglitazone [32]. In one of the patients, spirometry was measured before and after starting TZDs, and there was a significant improvement in both FVC and FEV_1_. A placebo controlled randomized study of 32 patients also found a modest decrease in late phase asthma reactivity to allergen challenge after four weeks of treatment with rosiglitazone [19]. A separate study of 16 asthmatic patients found a similar decrease in airway bronchoconstriction with methacholine challenge after 12 weeks of treatment with rosiglitazone [20]. Comparing rosiglitazone with inhaled corticosteroid treatment, a randomized trial of 46 patients with asthma showed improvement in FEV_1_ and FEF_25–75%_ among patients treated with rosiglitazone [21]. Our findings, using a much larger sample of patients, are consistent with these studies and demonstrate a positive impact of TZDs on asthmatic patients.

While there may be a beneficial effect of TZDs in asthma, it is also important to note TZDs have been associated with multiple adverse effects, including weight gain and heart failure, bladder cancer (in the case of pioglitzone) and possible cardiovascular effects (in the case of rosiglitazone) [33-35]. These adverse effects would have to be weighed against any potential gain in asthma management.

Limitations of this study include the non-randomized study design. Though it would also be desirable to perform a new-user study design in order to overcome biases with prevalent-user studies, we did not have a large enough sample size to evaluate new users of TZDs [36]. Furthermore, because these are observational analyses, we cannot infer causality in the association between TZDs and decreased asthma exacerbations, though this does add evidence to support their relationship. We attempted to control for a broad range of potential confounding factors, though unmeasured variables could have impacted the outcome. Notably, we did not have data on tobacco use or obesity, both of which are associated with asthma development and asthma exacerbations [37,38]. We also did not have information on spirometry or bronchoprovocation testing and could not adjust for the severity of asthma based on these measures. Prescriptions for respiratory medications and prior asthma exacerbations may be a marker of asthma severity and we included these variables in our multivariable analyses, though prescriptions for asthma medications is not a perfect proxy for severity [39].

Our study population comprised mostly elderly, male veterans, which may limit the generalizability of these findings. Incidence of asthma exacerbations in our study was low. This may be partly due to the population that we studied, as the risk of asthma exacerbation decreases with increasing age and with male gender [40]. In addition, our definition of exacerbations may not have adequately captured all patients experiencing an acute asthma attack. We broadened our analysis to assess the impact of TZDs on the risk for any oral steroid prescription and found similar results, though we could not ascertain the indication for these prescriptions. Despite these limitations, we were able to study a large cohort of asthmatic patients who were taking TZDs and control for a wide variety of potential confounding factors.

## Conclusion

Our findings suggest that TZDs could provide a novel approach for the prevention of asthma exacerbations among patients with diabetes. While there have been clinical studies on the association of TZDs with pulmonary function and allergen challenge, randomized trials are needed to study the role of TZDs in preventing asthma exacerbations. This may provide an innovative development in asthma management and help alleviate a growing healthcare burden.

## Abbreviations

CI: Confidence interval; IRR: Incidence rate ratio; OR: Odds ratio; PPARγ: Peroxisome proliferator-activated receptor gamma; TZD: Thiazolidinedione; VA: Veterans Affairs.

## Competing interests

Research funding, in part, was provided to DHA and CLB by Gilead Sciences. TGO is an employee of Gilead Sciences.

## Authors’ contributions

STR, DHA, TGO, CFL, LCF, BFC, and CLB were involved in the conception, hypotheses, and design of the study. MP, DHA, TGO, CLB, and CFL were involved in data collection. Data analysis and interpretation was performed by STR, CFL, DHA, LCF, and MP. All authors assisted with drafting and revisions. All authors approved the final version.
